# Effectiveness of tacrolimus therapy in refractory ulcerative colitis compared to infliximab with propensity score matching

**DOI:** 10.1038/s41598-024-77365-y

**Published:** 2025-01-02

**Authors:** Takeo Yoshihara, Takahiro Amano, Shinichiro Shinzaki, Yuri Tsujii, Akiko Asakura, Taku Tashiro, Mizuki Tani, Yuriko Otake-Kasamoto, Takuya Yamada, Yuko Sakakibara, Naoto Osugi, Shuji Ishii, Satoshi Egawa, Manabu Araki, Yuki Arimoto, Masanori Nakahara, Yoko Murayama, Ichizo Kobayashi, Kazuo Kinoshita, Hiroyuki Ogawa, Satoshi Hiyama, Narihiro Shibukawa, Masato Komori, Yorihide Okuda, Takashi Kizu, Tetsuhisa Kitamura, Minoru Kato, Yoshiki Tsujii, Takahiro Inoue, Hideki Iijima, Yoshito Hayashi, Tetsuo Takehara

**Affiliations:** 1 Department of Gastroenterology and Hepatology, Osaka University Graduate School of Medicine, 2-2 K1, Yamadaoka, Suita, Osaka 565-0871 Japan; 2 Division of Gastroenterology and Hepatology, Department of Internal Medicine, Hyogo Medical University, Nishinomiya, Japan; 3 Department of Gastroenterology and Hepatology, Osaka Rosai Hospital, Sakai, Japan; 4 Department of Gastroenterology, National Hospital Organization Osaka National Hospital, Osaka, Japan; 5 Department of Gastroenterology, Toyonaka Municipal Hospital, Toyonaka, Japan; 6 Department of Gastroenterology, Osaka General Medical Center, Osaka, Japan; 7 Department of Gastroenterology, Osaka Police Hospital, Osaka, Japan; 8 Department of Gastroenterology, National Hospital Organization Osaka Minami Medical Center, Kawachinagano, Japan; 9 Department of Gastroenterology, Kansai Rosai Hospital, Amagasaki, Japan; 10 Department of Gastroenterology, Ikeda City Hospital, Ikeda, Japan; 11 Department of Gastroenterology and Hepatology, Itami City Hospital, Itami, Japan; 12 Department of Gastroenterology, Higashiosaka City Medical Center, Higashiosaka, Japan; 13 Department of Gastroenterology, Otemae Hospital, Osaka, Japan; 14 Department of Gastroenterology, Nishinomiya Municipal Central Hospital, Nishinomiya, Japan; 15 Department of Gastroenterology, Japan Community Healthcare Organization Osaka Hospital, Osaka, Japan; 16 Department of Gastroenterology, Daini Osaka Police Hospital, Osaka, Japan; 17 Department of Gastroenterology, Hyogo Prefectural Nishinomiya Hospital, Nishinomiya, Japan; 18 Department of Gastroenterology, Saiseikai Senri Hospital, Suita, Japan; 19 Department of Gastroenterology, Yao Municipal Hospital, Yao, Japan; 20 Environmental Medicine and Population Sciences, Department of Social and Environmental Medicine, Graduate School of Medicine, Osaka University, Suita, Osaka Japan

**Keywords:** Propensity score matching analysis, Calcineurin inhibitors, Ulcerative colitis, Biomarkers, Medical research, Gastroenterology, Gastrointestinal diseases, Inflammatory bowel disease, Ulcerative colitis

## Abstract

There is insufficient evidence comparing the outcomes of tacrolimus-based remission induction therapy with infliximab in refractory ulcerative colitis (UC) and evidence regarding optimal strategies after tacrolimus-based remission induction therapy. We conducted a multi-institutional retrospective study of patients with UC treated with tacrolimus or infliximab between January 2010 and March 2019. The proportion of clinical remission at week 8 and cumulative colectomy-free rate were examined using propensity score matching analysis. The predictors for colectomy after tacrolimus induction were also investigated. Ninety patients in the tacrolimus group and 151 in the infliximab group were enrolled. The proportion of patients in clinical remission at week 8 was 65.2% in the matched tacrolimus group and 37.3% in the matched infliximab group (P = 0.0016), and the long-term colectomy-free rate was lower in the matched tacrolimus group than in the matched infliximab group (P = 0.0003). After clinical remission with tacrolimus, a serum albumin level of ≤ 3.5 g/dL at week 8 was extracted as a factor predicting colectomy (area under the curve: 0.94). Tacrolimus showed a higher remission induction effect for UC compared to infliximab. However, a high rate of colectomy after transition to maintenance treatment was found to be a concern for tacrolimus therapy.

## Introduction

Ulcerative colitis (UC) is a chronic inflammatory colon disease of unknown etiology presenting symptoms such as diarrhea, bloody stools, and abdominal pain, significantly reducing patients’ quality of life^1–3^. Treatment options for patients with steroid-dependent or -refractory UC are increasing, and favorable outcomes with advanced therapy such as biologics and small molecule drugs (SMDs) such as calcineurin inhibitors (CNIs) and janus kinase (JAK) inhibitors have been reported^4,5^. Conversely, surgery rates at 5 years after UC onset have been reported as 3–13%, 8.5–19% at 10 years, and 11–20% at 20 years^3^; therefore, induction and maintenance of remission to avoid surgery remain critical issues.

In patients who do not respond to intravenous steroid therapy or have a severe or acutely deteriorating disease course, potent immunosuppression with anti-tumor necrosis factor (TNF) agents or CNIs should be used to avoid surgery^6,7^. Two types of CNIs, cyclosporine^8^, and tacrolimus^9,10^, are used in remission induction therapy for moderate-to-severe UC. Tacrolimus exerts a potent immunosuppressive effect by inhibiting T-cell activation and proliferation, with greater immunosuppressive activity than cyclosporine^11^. Tacrolimus is better absorbed in the intestinal tract than cyclosporine and can be administered orally^12^. Among CNIs, only tacrolimus has been approved for clinical use in Japan for remission induction in UC^13–15^.

Although two randomized controlled trials (RCTs) comparing the efficacy of cyclosporine and infliximab have reported similar clinical remission proportions and colectomy rates in severe UC^16,17^, two meta-analyses on colectomy rates for cyclosporine and infliximab have reported differing results. Barberio et al. reported similar colectomy rates in cyclosporine and infliximab groups^18^. In contrast, Jia et al.^19^ reported significantly lower surgical rates (short-term and within 1 year) in the infliximab group than that in the cyclosporine group. However, no RCT has been conducted to compare tacrolimus with infliximab regarding treatment efficacy and colectomy rates. Notably, several retrospective observational studies have shown comparable efficacy and colectomy rates of tacrolimus and infliximab in moderate-to-severe UC^7,20–24^. Furthermore, most observational studies did not adjust for background factors in the tacrolimus and infliximab groups. Therefore, the results of these retrospective studies should be interpreted with caution. Two meta-analyses that included a few observational studies on the efficacy and surgical rates of tacrolimus and infliximab reported that tacrolimus therapy was comparable with infliximab therapy^19,25^. Thus, the difference between tacrolimus and infliximab regarding the efficacy of remission induction therapy for steroid-dependent and -refractory UC still needs clarification.

Furthermore, there is insufficient evidence for remission maintenance therapy in patients who have achieved remission with tacrolimus^10,26^. Immunomodulators (IM) such as thiopurines are often used as maintenance therapy after remission with tacrolimus^11,26–28^; however, the optimal strategy for maintenance therapy after remission has been induced by tacrolimus, whether IM or advanced therapy should be selected, is not well understood^29^. The efficacy and safety of maintenance therapy with IM for UC is increasingly debated, with renewed interest in its use regarding efficacy and cost^30,31^. The medical costs associated with inflammatory bowel disease (IBD) are increasing with the growing use of biologics and SMDs, and treatment strategies to avoid overtreatment are necessary for sustainable healthcare economics^2,32^. Identifying the risk factors for relapse and colectomy after remission induction with tacrolimus contributes to the appropriate use of tacrolimus and biologics and can lead to cost savings^29,31^.

Therefore, there is a need for more evidence regarding the therapeutic efficacy of tacrolimus and infliximab for the optimal and cost-effective treatment of refractory UC. This multi-institutional study aimed to investigate the induction and maintenance of remission and the colectomy rates with tacrolimus and infliximab in patients with refractory UC.

## Materials and methods

### Study design and patients

This was a retrospective multi-institutional study. Patients aged at least 16 years who were diagnosed with refractory UC (corticosteroid refractory or dependence) following the Japanese IBD guideline^13,14^ and initiated on tacrolimus or infliximab between January 2010 and March 2019 at 18 hospitals participating in the Osaka Gut Forum were eligible for analysis. Patients who underwent a colectomy before treatment were excluded. The study was conducted in accordance with relevant guidelines/regulations including the Declaration of Helsinki, and the requirement for written informed consent was waived by allowing the participants to opt out because of the study’s retrospective nature. This study was approved by the Ethics Committee of Osaka University Hospital and by the Ethics Committees of the respective institutions (Supplementary Table 1, the ethics approval number: 18208).

### Data collection

Patient characteristics at the initiation of treatment included sex, age, duration of disease, body mass index (BMI), smoking history, previous induction of remission with steroids, previous use of biologics and SMDs, the extent of the disease, and information on the use of IM, 5-aminosalicylates (5-ASA), and steroids. Disease severity was classified using the Mayo score (mild: 3–5, moderate: 6–10, severe, > 11)^33,34^. Clinical features such as partial Mayo scores at 8 (± 4) and 52 (± 8) weeks after treatment initiation were also obtained. Clinical remission was defined as a partial Mayo score of 2 and no individual sub-score > 1. We extracted information on colectomy and adverse events that required discontinuation of tacrolimus or infliximab through August 31, 2019.

### Treatment

Patients received either tacrolimus or infliximab as remission induction therapy for UC upon admission. The physicians were free to decide on the treatment according to the patient’s condition, wishes or physician’s preference. As the study only enrolled patients whose treatment was initiated since 2010, treatment was chosen in an environment where both drugs were available to almost all patients. In the tacrolimus group, approximately 0.05 mg/kg tacrolimus was administered twice daily as an initial dose. Dose adjustments were implemented to achieve blood concentrations within the high-trough therapeutic range (10–15 ng/mL). After a two-week high-trough period, tacrolimus blood concentrations were adjusted to be within the low-trough therapeutic range (5–10 ng/mL). Since Japanese medical insurance stipulates that tacrolimus should be used for remission induction therapy, patients who achieved clinical remission received maintenance therapy, switching to thiopurine or biologic therapy^15^. Each institution’s attending physician decided the treatment strategy after achieving remission with tacrolimus. In the infliximab group, the patients received remission induction and maintenance therapy with infliximab using the following protocol: 5 mg/kg of infliximab was administered as an intravenous induction regimen at week 0, 2, and 6, followed by a maintenance regimen of 5 mg/kg every 8 weeks. Due to the restriction by the Japanese medical insurance system at the time, dose intensification and interval shortening of infliximab were not performed.

### Statistical analysis

Continuous variables were summarized using median values and interquartile ranges and analyzed using the Wilcoxon rank-sum test. Categorical variables were described as proportions and analyzed using Fisher’s exact or chi-squared test. The time to colectomy and adverse events were examined using Kaplan–Meier curves and tested using the log-rank test. Univariate Cox proportional hazards regression was performed to identify the factors significantly affecting the outcome. The multivariate Cox proportional hazards regression model included all factors with a p-value < 0.05. Propensity score matching between tacrolimus and infliximab therapies was implemented in a 1:1 ratio using nearest neighbor matching with a 0.2 calliper. We examined balanced variable distribution after matching with a threshold of absolute standardized differences < 0.25^35,36^. The receiver operating characteristic (ROC) curve was applied to set up the cutoff value and area under the curve (AUC), and Akaike’s information criterion (AIC) was calculated to compare predictability^37,38^. In these examinations, statistical significance was set at a p-value < 0.05. We used the available-case analysis to deal with the missing data. Statistical analyses were performed using JMP Pro statistical software (version 15.2.0; SAS Institute, Inc., Cary, NC, USA) and EZR version 1.61 (Saitama Medical Center, Jichi Medical University, Saitama, Japan)^39^.

## Results

### Patient characteristics

In total, 241 patients were enrolled in the study: 90 in the tacrolimus group and 151 in the infliximab group (Table 1). The ratio of tacrolimus and infliximab use remained almost constant during the enrolment period from 2010 to 2019 except 2012 (Supplementary Fig. 1). The median duration of tacrolimus therapy was 3 months (interquartile range: 1–5) and infliximab therapy was 14 months (interquartile range: 3–43). The tacrolimus group had a significantly higher partial Mayo score and a significantly higher Mayo-based severity classification, but the Mayo endoscopic subscore was similar in both groups. Regarding blood markers, the serum C-reactive protein (CRP) level was significantly higher, and the serum albumin level was significantly lower in the tacrolimus group than that in the infliximab group. These results suggested that UC disease activity was higher in the tacrolimus group than that in the infliximab group.

**Table 1:** Patient characteristics.

	Tacrolimus group N = 90	Infliximab group N = 151	P value
Male, n (%)	53 (58.9)	91 (60.3)	n.s.
Age (y), median [IQR]	44 [33–58]	46 [30–61]	n.s.
Disease duration (y), median [IQR]	2.5 [0–8.3]	2.0[1.0–7.0]	n.s.
BMI (kg/m^2^), median [IQR]	20.2 [18.0–22.6]	20.2 [18.0–22.6]	n.s.
Smoking, n (%)	21 (23.3)	36 (24.0)	n.s.
History of induction with steroids, n (%)	38 (42.2)	80 (53.0)	n.s.
Biologic/SMD naive	69 (76.7)	112 (74.2)	n.s.
Concomitant medications			
5-ASA, n (%)	85 (94.4)	138 (91.4)	n.s.
Steroids, n (%)	59 (65.7)	92 (60.9)	n.s.
Thiopurines, n (%)	21 (23.3)	68 (45.0)	0.0006
Disease phenotype (proctitis/left-sided/pan-colitis), n	0/19/71	2/43/106	n.s.
Partial Mayo score, median [IQR]	7 [6–8]	5 [4–7]	< 0.0001
Mayo endoscopic subscore, (1/2/3)	3/34/46	16/64/53	n.s.
Disease severity (Mild/Moderate/Severe)	6/60/24	41/87/23	0.0002
Hemoglobin (g/dl)	10.7 [9.7–12.6]	11.4 [10.1–12.9]	n.s.
CRP (mg/dl), median [IQR]	3.4 [1.0–6.1]	0.7 [0.1–2.9]	< 0.0001
Albumin (g/dl), median [IQR]	2.9 [2.3–3.5]	3.4 [2.8–3.9]	< 0.0001

IQR, interquartile range; BMI, body mass index; SMD, small molecule drug; ASA, aminosalicylic acid; CRP, c-reactive protein; n.s., not significant.

### Clinical efficacy and colectomy-free rate

**Fig. 1:**
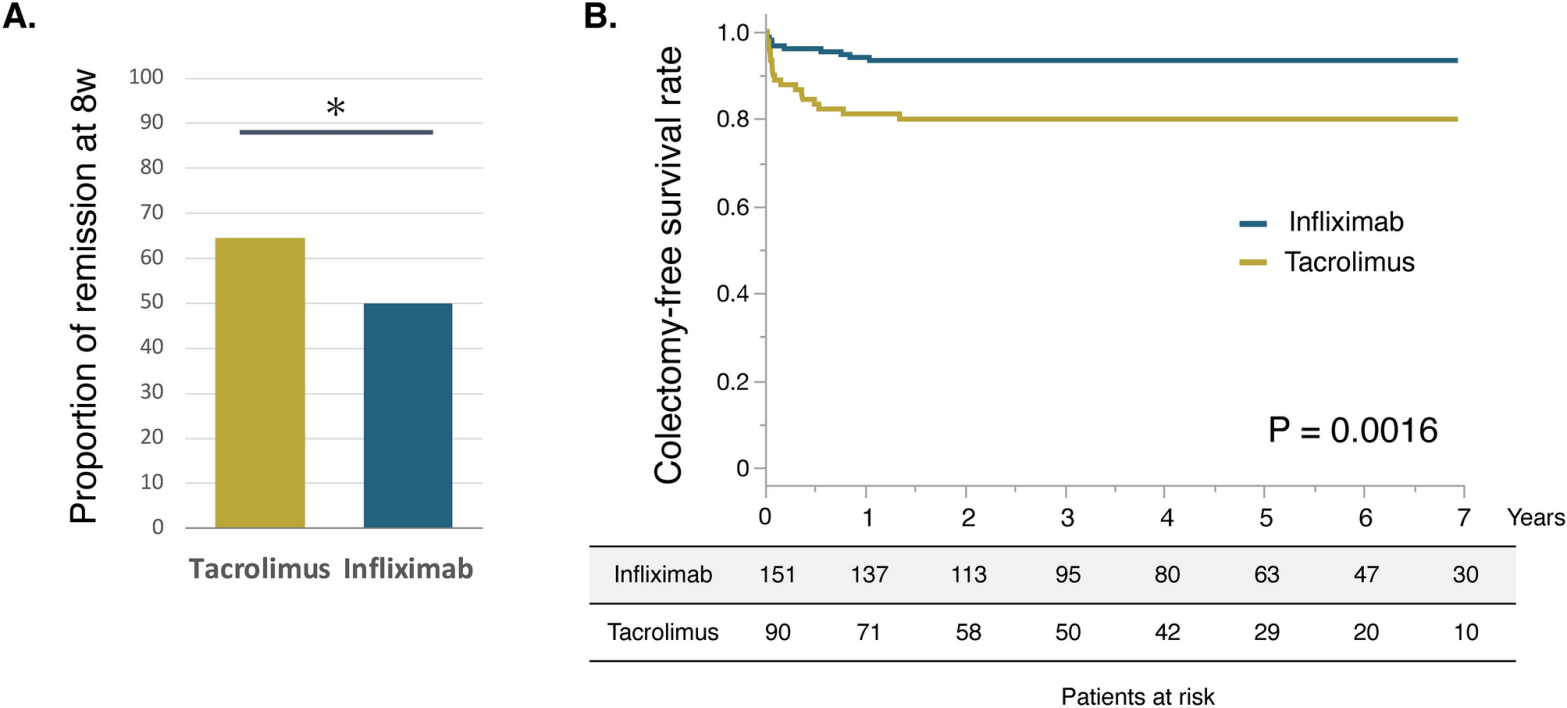
Proportion of clinical remission and cumulative colectomy-free rate in the tacrolimus and infliximab groups among the pre-matched cohort. (**A**) The proportion of clinical remission at week 8. (**B**) Kaplan–Meier curve for the colectomy-free survival rate in the tacrolimus group vs infliximab group. * P < 0.05.

The proportion of patients who achieved clinical remission at week 8 was significantly higher in the tacrolimus group than that in the infliximab group, despite higher UC disease activity in the tacrolimus group (tacrolimus group, 64.4%; infliximab group, 50.0%; P = 0.0226; Fig. 1A). We also assessed long-term prognosis regarding colectomy. The median observation period was 1185 days (interquartile range: 592–1940), and colectomy was performed in 29 patients (12.0%). Kaplan–Meier curves showed that the cumulative colectomy-free rate was significantly lower in the tacrolimus group than that in the infliximab group (Fig. 1B; P = 0.0016; log-rank test). We then performed a subgroup analysis, dividing patients by severity (moderate/severe) based on the Mayo score. The tacrolimus group showed a higher proportion of patients who achieved clinical remission at week 8 than the infliximab group in subgroup of patients with moderately active UC (Tacrolimus group, 67.2%; Infliximab group, 41.8%; P = 0.0032; Supplementary Fig. 2A) and showed no significant difference between the tacrolimus and infliximab groups in the proportion of colectomy within 12 weeks (tacrolimus group: 6.7%, infliximab group: 6.9%, P = 0.9566; Supplementary Fig. 2B). In the long-term analysis regarding colectomy, there was no significant difference in the colectomy-free rate between the tacrolimus group and infliximab group in the subgroup analysis of patients with moderately active UC (P = 0.1916; log-rank test; Supplementary Fig. 2C). The subgroup analysis in the patients with severely active UC showed a similar trend of the proportion of clinical remission at week 8 (Tacrolimus group 66.7%, Infliximab group 39.1%, P = 0.0586, Supplementary Fig. 2A). In the analysis of colectomy, the proportion of colectomy at week 12 and the long-term colectomy-free rate in the tacrolimus group tend to be higher than in the infliximab group (colectomy at week 12: Tacrolimus group: 20.8%, Infliximab group 8.7%, P = 0.2427, Supplementary Fig. 2B; long-term colectomy-free rate: P = 0.0517, log-rank test, Supplementary Fig. 2D). In these subgroup analyses (moderate/severe), UC disease activity was also higher in the tacrolimus group than that in the infliximab group (Supplementary Tables 2, 3).

### Factors associated with colectomy

Univariate Cox proportional hazards regression analyses were performed to investigate confounding factors for the colectomy outcome (Table 2). Continuous variables reflecting disease severity, such as CRP and albumin levels, were categorized using the median value of the tacrolimus group as the cutoff value. Disease duration > 3 years, BMI > 20 kg/m^2^, history of induction therapy with steroids, disease phenotype, serum CRP level > 3.4 mg/dL, and serum albumin level ≤ 2.9 g/dL were significantly associated with colectomy risk. The risk ratio (RR) of colectomy in the tacrolimus group was significantly higher than that in the infliximab group (RR = 3.18; 95% CI, 1.43–7.10; P = 0.0047; Table 2). We also conducted a multivariate Cox regression analysis using all variables significantly associated with colectomy in the univariate analyses. Multivariate analysis showed that the RR of colectomy in the tacrolimus group was significantly higher than that in the infliximab group (adjusted RR = 2.58; 95% CI, 1.02–6.50; P = 0.0453; Table 2). Variables such as BMI > 20 kg/m^2^ and serum CRP level > 3.4 mg/dL were also significantly associated with colectomy risk after adjusting for the confounders mentioned above.

**Table 2:** Univariate and multivariate analysis for the colectomy.

	Univariate analysis		Multivariate analysis	
	RR [95% CI]	P value	RR [95% CI]	P value
Male	1.19 [0.61–2.34]	n.s.		
Age > 60 years old	1.40 [0.70–2.84]	n.s.		
Disease duration < 3 years	2.62 [1.32–5.20]	0.0057	1.84 [0.71–4.73]	n.s.
BMI < 20 kg/m^2^	2.22 [1.11–4.43]	0.0249	2.92 [1.10–7.83]	0.0322
Smoking	0.75 [0.33–1.69]	n.s.		
History of induction with steroids	2.37 [1.19–4.75]	0.0119	1.57 [0.58–4.25]	n.s.
Biologic/SMD naive	0.54 [0.28–1.05]	n.s.		
Concomitant medications				
5-ASA	1.21 [0.41–3.59]	n.s.		
Steroids	1.45 [0.72–2.91]	n.s.		
Thiopurines	0.87 [0.44–1.72]	n.s.		
Disease severity (Severe)	1.63 [0.71–3.74]	n.s.		
Disease phenotype (pan-colitis)	3.47 [1.20–10.0]	0.0214	3.03 [0.75–12.2]	n.s.
Hemoglobin (10.5 mg/dl)	2.07 [0.93–4.60]	n.s.		
CRP > 3.4 mg/dl	3.89 [1.98–7.67]	< 0.0001	3.30 [1.24–8.80]	0.0164
Albumin ≤ 2.9 g/dl	3.11 [1.58–6.10]	0.0010	2.34 [0.86–6.32]	n.s.
Tacrolimus	3.18 [1.43–7.10]	0.0047	2.58 [1.02–6.50]	0.0453

RR, risk ratio; IQR, interquartile range; BMI, body mass index; SMD, small molecule drug; ASA, aminosalicylic acid; CRP, c-reactive protein; n.s., not significant.

### Propensity score-matched analysis

**Fig. 2:**
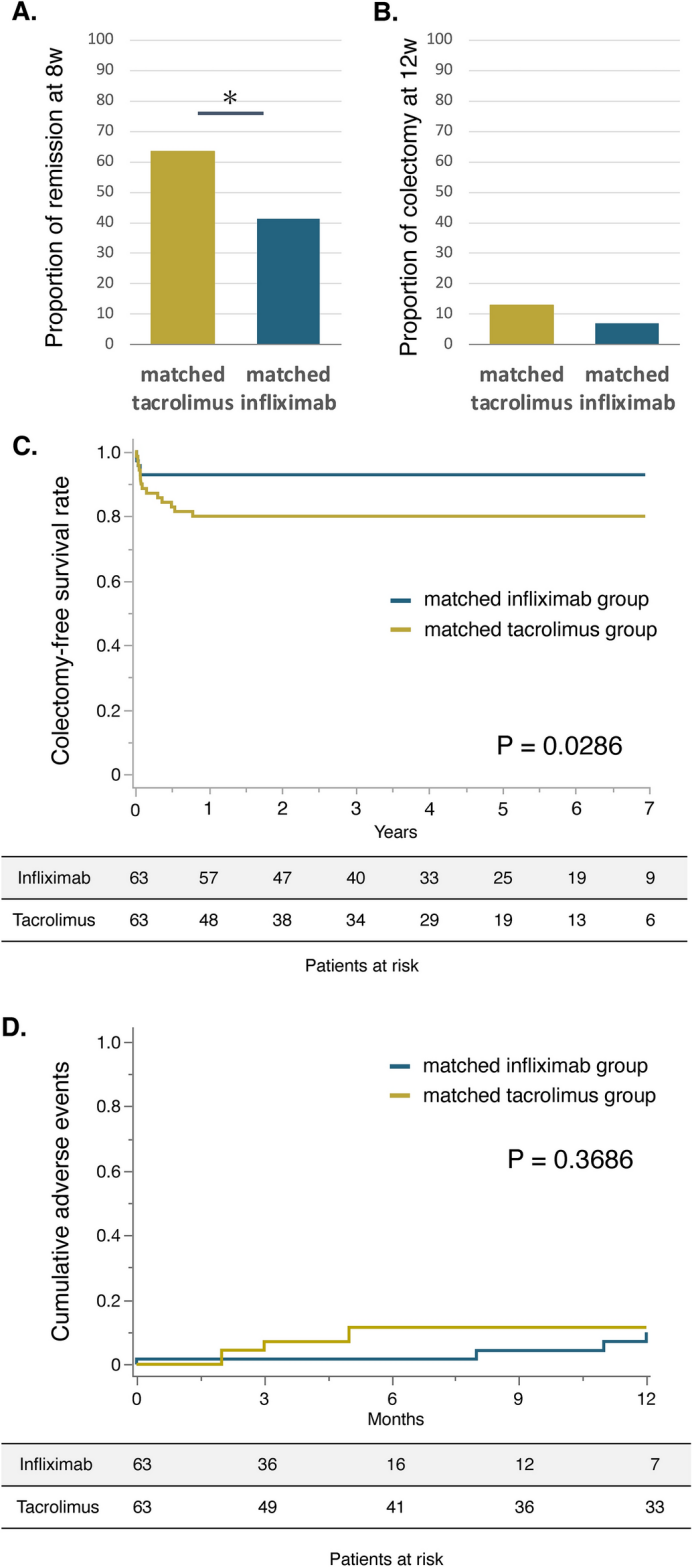
The efficacy and safety of tacrolimus therapy and infliximab therapy in the propensity score matching cohort. (**A**) The proportion of clinical remission at week 8. (**B**) The proportion of colectomy at week 12. (**C**) Cumulative colectomy-free survival rate in the matched tacrolimus group versus matched infliximab group. (**D**) Cumulative adverse events rate in the matched tacrolimus group versus matched infliximab group. * P < 0.05.

As the tacrolimus group had more severe UC disease activity than the infliximab group, we conducted a propensity score-matched analysis. We estimated a propensity score by fitting a logistic regression model that was adjusted for sixteen variables listed in Table 1 before the decision to administer tacrolimus or infliximab. The area under the ROC curve of the logistic-regression model to calculate a propensity score was 0.738. In this analysis, 63 patients in the infliximab group (matched infliximab group) were matched with 63 patients in the tacrolimus group (matched tacrolimus group). After propensity score matching, the patient characteristics were balanced between the matched tacrolimus and matched infliximab groups (Table 3). After matching, the proportion of patients in clinical remission at week 8 was significantly higher in the matched tacrolimus group than that in the matched infliximab group (matched tacrolimus group, 63.5%; matched infliximab group, 41.3%; P = 0.0125; Fig. 2A). There was no significant difference between the matched tacrolimus and matched infliximab groups in the proportion of colectomy within 12 weeks (tacrolimus group: 12.9%, infliximab group: 7.1%, P = 0.2598; Fig. 2B). In the long-term analyses, colectomy was performed in 14 cases (22.2%) in the matched tacrolimus group and six cases (9.5%) in the matched infliximab group during the observational period (1084 days [median, IQR 314–1929] in the matched tacrolimus group and 1586 days [median, IQR 693–2328] in the matched infliximab group). The cumulative colectomy-free rate was significantly lower in the matched tacrolimus group than that in the matched infliximab group (P = 0.0286, log-rank test, Fig. 2C). The cumulative colectomy-free rate in the matched tacrolimus group at 6 months and 12 months was 80.9% and 79.4%, respectively. Additionally, the cumulative colectomy-free rate in the matched infliximab group at 6 months and 12 months was 93.7% and 93.7%, respectively (Supplementary Fig. 3).

**Table 3:** Patient characteristics of propensity score matched tacrolimus group and matched infliximab group.

	Matched tacrolimus group	Matched infliximab group	P value	Standardized difference	
	n = 63	n = 63		After matching	Before matching
Male, n (%)	38 (60.3)	38 (60.3)	n.s.	< 0.001	0.028
Age (y), median [IQR]	41 [31–58]	42 [29–56]	n.s.	0.054	0.039
Disease duration (y), median [IQR]	3.0 [0.5–8.0]	3.0 [1.0–7.5]	n.s.	0.110	0.015
BMI (kg/m^2^), median [IQR]	19.9 [17.9–22.6]	20.4 [18.0–22.8]	n.s.	0.178	0.069
Smoking, n (%)	13 (20.6)	15 (23.8)	n.s.	0.076	0.016
History of induction with steroids	27 (42.9)	27 (42.9)	n.s.	< 0.001	0.217
Biologic/SMD naïve	45 (71.4)	42 (66.7)	n.s.	0.032	0.103
Concomitant medications					
5-ASA, n (%)	58 (92.1)	60 (95.2)	n.s.	0.130	0.119
Steroids, n (%)	42 (66.7)	39 (61.9)	n.s.	0.100	0.096
Thiopurines, n (%)	17 (27.0)	16 (25.4)	n.s.	0.036	0.470
Mayo endoscopic subscore (1/2/3)	4/27/30	5/22/31	n.s.	0.138	0.343
Partial Mayo score	7 [5.5–7]	6 [5–8]	n.s.	0.062	0.644
Severity (severe)	11 (17.5)	16 (25.4)	n.s.	0.194	0.594
Disease phenotype (pancolitis)	50 (79.4)	48 (76.2)	n.s.	0.076	0.200
CRP (mg/dl)	2.26 [0.48–5.03]	1.36 [0.26–5.00]	n.s.	0.224	0.503
CRP > 3.4 mg/dl	34 (54.0)	41 (65.1)	n.s.	0.228	0.559
Hemoglobin (mg/dl)	10.9 [9.2–12.8]	10.7 [9.7–12.0]	n.s.	0.224	0.209
Hemoglobin (< 10.5 mg/dl)	37 (58.7)	38 (60.3)	n.s.	0.228	0.295
Albumin (g/dl)	3.1 [2.3–3.6]	3.2 [2.4–3.6]	n.s.	0.052	0.520
Albumin ≤ 2.9 g/dl	28 (44.4)	26 (41.3)	n.s.	0.064	0.513

IQR, interquartile range; BMI, body mass index; SMD, small molecule drug; ASA, aminosalicylic acid; CRP, c-reactive protein; n.s., not significant.

### Adverse events

In the pre-matched patients, 21 adverse events that required discontinuation of tacrolimus or infliximab therapy occurred (five patients in the tacrolimus group and 16 patients in the infliximab group). Among the adverse events, 9 infusion reactions were observed only in the infliximab group, and the remaining 12 cases were due to toxic events other than infusion reactions. After propensity score matching, there was no significant difference in the cumulative rate of adverse events within 1 year between the matched tacrolimus and matched infliximab groups (matched tacrolimus group, 8.0%; matched infliximab group, 18.5%; P = 0.3686, log-rank test, Fig. 2D).

### Maintenance therapy after achieving clinical remission with tacrolimus therapy

**Fig. 3:**
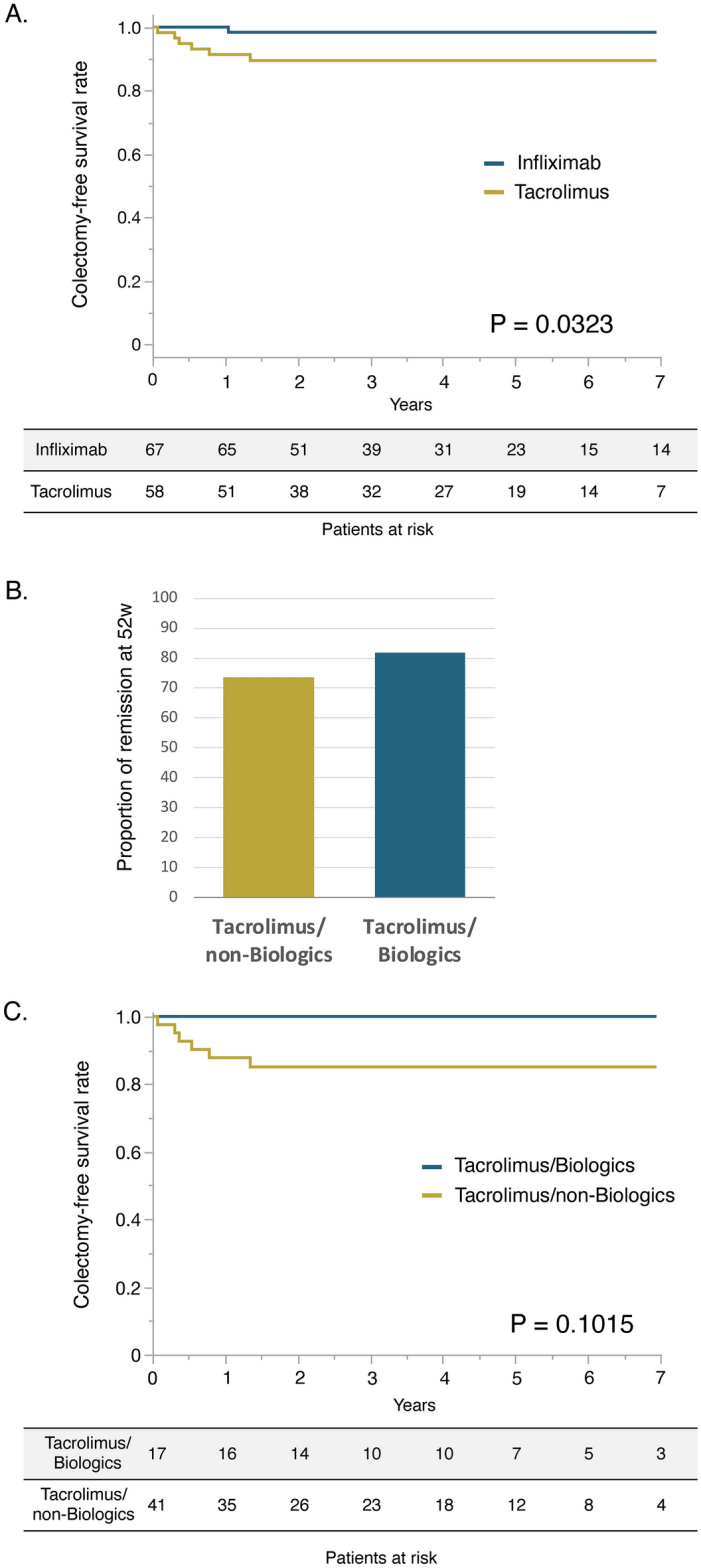
(**A**) Cumulative colectomy-free survival rate in patients who achieved clinical remission at week 8 after treatment initiation in the tacrolimus group versus infliximab group. (**B**) The proportion of remission maintenance at week 52 in patients achieving remission at week 8 in the tacrolimus/non-Biologics group versus tacrolimus/biologics group. (**C**) Cumulative colectomy-free survival rate in patients who achieved remission at week 8 after treatment initiation in the tacrolimus/non-biologics group versus tacrolimus/biologics group.

Next, we investigated the factors associated with colectomy in patients who achieved clinical remission at week 8. When comparing the cumulative colectomy-free rates in patients who achieved remission at week 8 after treatment initiation in both groups (tacrolimus group, 58; infliximab group, 67), the cumulative colectomy-free rate was significantly lower in the tacrolimus group than that in the infliximab group (Fig. 3A, P = 0.0323, log-rank test). Forty-one of the 58 patients in the tacrolimus group who successfully achieved remission induction were on maintenance therapy with IM or 5-ASA (tacrolimus/non-biologics group), whereas 17 patients were consecutively treated with biologics (infliximab: 10, adalimumab: three, golimumab: two, vedolizumab: two) after tacrolimus as maintenance therapy (tacrolimus/biologics group). The partial Mayo score at week 8 was significantly higher in the tacrolimus/biologics group than that in the tacrolimus/non-biologics group. There were no significant differences in patient characteristics or duration of tacrolimus therapy between the tacrolimus/non-biologics and tacrolimus/biologics groups (Supplementary Table 4). The proportions of remission at week 52 were 73.5% in the tacrolimus/non-biologics group and 81.8% in the tacrolimus/biologics group (Fig. 3B, P = 0.6782). The tacrolimus/non-biologics group had a colectomy rate of 14.6% (6/41), whereas that of the tacrolimus/biologics group was 0% (0/17). The cumulative colectomy rate tended to be higher in the tacrolimus/non-biologics group than that in the tacrolimus/biologics group; however, the difference was insignificant (Fig. 3C, P = 0.1015, log-rank test). Next, factors contributing to colectomy in the tacrolimus/non-biologics group were examined. There were no significant differences in the characteristics at week 0 between patients with and without colectomy (Supplementary Table 5). When comparing disease activity monitoring markers at week 8, the serum CRP level, albumin level, and partial Mayo score at week 8 significantly differed between patients with and without colectomy (Table 4). The cutoff values to predict colectomy were calculated using ROC curves: CRP: 0.27 mg/dL (AUC: 0.79, AIC: 34.5), albumin: 3.5 g/dL (AUC: 0.94, AIC: 21.8), and partial Mayo score: 2 (AUC: 0.71, AIC: 33.6). Serum albumin level at week 8 showed the highest AUC and lowest AIC, suggesting that it is the most helpful cutoff value for predicting colectomy. Univariate analyses were performed using these cutoff values. The results revealed that serum CRP and albumin values at week 8 were associated with colectomy (Table 4). A multivariate analysis was not performed due to the small sample size.

**Table 4:** Patient characteristics, ROC and univariate analysis for colectomy at week 8 between patients with or without colectomy in the tacrolimus/non-Biologics group.

	Patient characteristics			ROC analysis		Univariate analysis	
	Colectomy	Colectomy-free	P value	AUC	P value	OR [95% CI]	P value
CRP at week 8 (mg/dl), median [IQR]	0.44 [0.22–1.50]	0.09 [0.04–0.21]	0.0238				
CRP at week 8 > 0.27 mg/dl	4 (67)	7 (21)	0.0423	0.79	n.s	5.42 [1.15–25.5]	0.0323
Albumin at week 8 (g/dl), median [IQR]	3.4 [3.0–3.7]	4.1 [3.9–4.4]	0.0006				
Albumin at week 8 ≤ 3.5 g/dl	5 (83)	2 (7)	0.0002	0.94	< 0.0001	15.0 [2.74–82.2]	0.0018
partial Mayo score at week 8 (0/1/2)	2/0/4	18/11/4	0.0074				
partial Mayo score at week 8 = 2	4 (67)	4 (12)	0.0105	0.71	0.0394	4.10 [0.88–19.1]	n.s

ROC, receiver operating curve; AUC, area under the curve; OR, odds ratio; CI, confidence interval; CRP, c-reactive protein; IQR, interquartile range; n.s., not significant.

## Discussion

This multicenter observational study, conducted on the largest scale ever, provides crucial evidence regarding the effectiveness of tacrolimus and infliximab in refractory UC. To our knowledge, this is the first study to compare the effectiveness of tacrolimus and infliximab in patients with UC using propensity score matching. This study’s key findings are the higher proportion of remission in the induction phase and colectomy rate in the long-term analysis with tacrolimus than that with infliximab agents using propensity score matching analysis, the factors contributing to colectomy after successful induction of remission with tacrolimus, and the effectiveness of non-advanced therapy as remission maintenance treatment after remission induction with tacrolimus.

Notably, CNIs and infliximab are useful and equally effective in inducing remission in moderate to severe UC^18,19^. Furthermore, most studies comparing CNIs and infliximab have reported on cyclosporine^16,17^; accordingly, evidence comparing the usefulness of tacrolimus and infliximab is still required. This study showed that tacrolimus therapy produced a higher proportion of remission induction after 8 weeks than that of infliximab therapy, and the colectomy-free rate was lower in the tacrolimus group than that in the infliximab group. Multivariate analysis of factors associated with colectomy rate included low BMI, high serum CRP levels, and tacrolimus treatment as independent factors. Propensity score matching allowed us to balance the background factors of the tacrolimus and infliximab groups. After matching, the proportion of remission induction was higher in the matched tacrolimus group than that in the matched infliximab group, whereas the colectomy-free rate was significantly lower. In addition, adverse events requiring drug discontinuation were similarly low in the tacrolimus and infliximab groups, suggesting that both drugs can be used safely. Our study adjusted for confounding factors using propensity score matching; however, we could not investigate the effectiveness of tacrolimus and infliximab in each disease activity (severe/moderate) of UC using propensity score matching due to the insufficient number of patients.

The advantages of tacrolimus therapy for UC are its high remission-inducing effect and its low medication cost (approximately one-quarter of the cost of infliximab during the remission induction phase)^31^. Its disadvantages are its narrow therapeutic range and adverse events such as renal, cardiac, and neurological disorders associated with long-term use^11^. There is insufficient data on the efficacy and safety of long-term tacrolimus use^14^; therefore, Japanese medical insurances limit its use for remission induction therapy in patients with UC. Maintaining remission after remission induction with tacrolimus is still a crucial issue in tacrolimus therapy for patients with UC^15^. The efficacy of remission induction with CNIs, including tacrolimus, followed by remission maintenance with biologics, has been reported to compensate for this limitation. Pellet et al.^40^ reported the efficacy of remission induction with CNIs (cyclosporine: 37 cases, tacrolimus: two cases) followed by maintenance therapy with vedolizumab in steroid-refractory/-dependent UC. The American College of Gastroenterology clinical guideline recommends using vedolizumab and thiopurine as maintenance therapy after remission induction in acute severe UC (conditional recommendation, very low-quality evidence)^41^. This study also found a trend towards lower operative rates in the tacrolimus/biologics group, suggesting the use of biologics. However, the remission maintenance rate at week 52 in the tacrolimus/non-biologics group was as high as 73.5% (Fig. 3C), implying that biologics were unnecessary in many cases. In addition, the results suggested that serum CRP and albumin levels at week 8 were associated with the risk of colectomy for remission induction rather than background factors at the initiation of tacrolimus treatment. In particular, our results revealed that serum albumin level ≤ 3.5 g/dL at week 8 was the most predictive factor for colectomy. These results indicate that evaluation at week 8 helps select maintenance therapy in patients with successful remission induced by tacrolimus.

Induction of remission with tacrolimus and maintenance of remission with thiopurines instead of biologics and SMDs is an effective and preferable treatment from a medical-economic point of view^29^. Tacrolimus/IM therapy is a useful option, especially for patients who cannot use the advanced therapy due to health insurance systems, drug costs, and regionality. We have shown that serum albumin level at week 8 after tacrolimus induction is a valuable biomarker for predicting surgery. Therefore, we propose a strategy for remission maintenance therapy (biologics or IM) based on albumin levels at week 8 in patients who achieve clinical remission after tacrolimus induction. However, further studies are required to evaluate the appropriateness of this strategy.

The limitations of this study are as follows: First, this study had a selection bias and limited information acquisition due to its retrospective observational nature. We could not investigate the outcomes stratified by steroid-refractory or dependence due to a lack of information. Second, propensity score matching does not allow adjustment for unmeasurable or unmeasured confounders. In particular, items related to the diagnosis of acute severe UC (fever and tachycardia) was not obtained due to the nature of the retrospective study. Third, the study of tacrolimus and infliximab in patients with severe UC was underpowered. Forth, tacrolimus dose reduction/discontinuation protocols are not standardized, and the selection of maintenance therapy after remission induction with tacrolimus were also left to each institution’s attending physicians. Fifth, endoscopic evaluation and the data of disease activity monitoring markers such as fecal calprotectin and leucine-rich alpha-2 glycoprotein are unavailable^42^. Finally, data on emerging drugs such as non-anti-TNF agents and JAK inhibitors were not presented, and further studies are warranted.

We examined post-remission outcomes with tacrolimus treatment, which has rarely been reported. We proposed a treatment strategy which allows appropriate patient monitoring with tacrolimus and may contribute to reducing healthcare costs by avoiding the overuse of biologics. Furthermore, it is difficult to conduct RCTs comparing tacrolimus and infliximab in the current situation, where many available biologics/SMD exist. This study substitutes for an RCT, which is not feasible and has high external validity by using real-world data that cannot be obtained in an RCT. Here, we present valuable evidence and therapeutic strategies for treating refractory UC.

In this study, the effectiveness of tacrolimus therapy in inducing remission was found to be higher compared to infliximab therapy. However, during the induction phase, tacrolimus did not reduce the need for colectomy and actually led to a higher rate of colectomy during the maintenance phase. These results suggested that the seamless transition from the induction phase to the maintenance phase of infliximab contributed to a long-term reduction in the rate of colectomy, and showed the significance of the selection of maintenance therapy after remission induction with tacrolimus. In conclusion, it could be beneficial to consider an early transition to biologic maintenance therapy to capitalize on the high remission induction effect of tacrolimus. Additionally, the levels of albumin after remission induction could serve as a useful biomarker for determining the switch to biologic therapy.

## Supplementary Information

Supplementary Information 1.

Supplementary Information 2.

Supplementary Information 3.

Supplementary Information 4.

## Data Availability

The data supporting this study’s findings can be requested from the corresponding author. The data are not publicly available due to restrictions containing information that could compromise the privacy of research participants.

## Abbreviations

TNF: Tumor necrosis factor
UC: Ulcerative colitis
CNI: Calcineurin inhibitors
JAK: Janus kinase
SMD: Small molecule drugs
RCT: Randomized controlled trials
IM: Immunomodulators
IBD: Inflammatory bowel disease
BMI: Body mass index
5-ASA: 5-Aminosalicylic acid
ROC: Receiver operating characteristic
AUC: Area under the curve
AIC: Akaike’s information criterion
CRP: C-reactive protein
