# Supplementary figures and images for: Effectiveness of tacrolimus therapy in refractory ulcerative colitis compared to infliximab with propensity score matching
Source: Sci Rep. 2025 Jan 2;15:68. doi: 10.1038/s41598-024-77365-y (PMC11696101; doi:10.1038/s41598-024-77365-y)

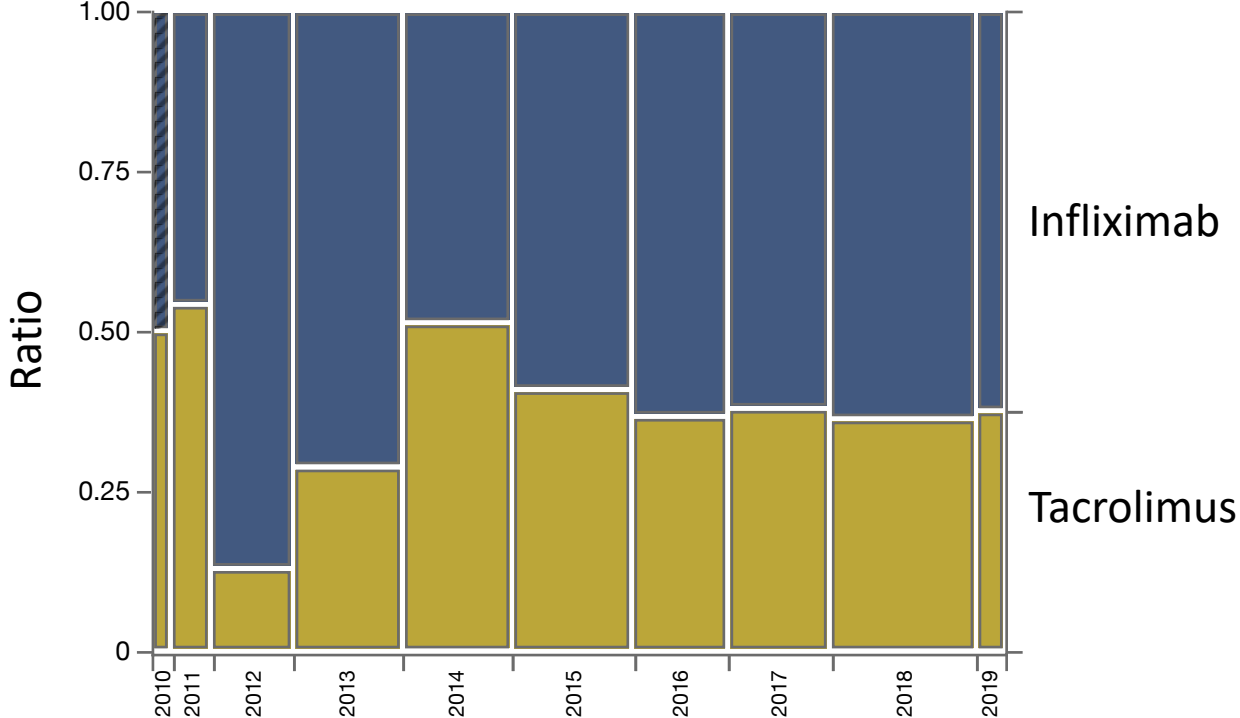

**Supplementary Figure 1.** Ratio of tacrolimus and infliximab use during the enrollment period.

Supplement: Supplementary file 1 — Supplementary Information 1. [file 41598_2024_77365_MOESM1_ESM.pdf]

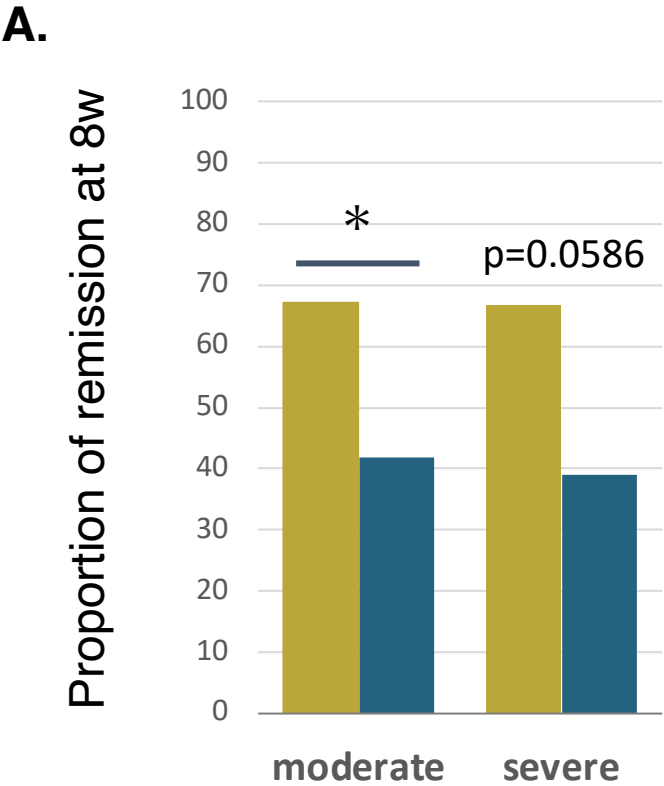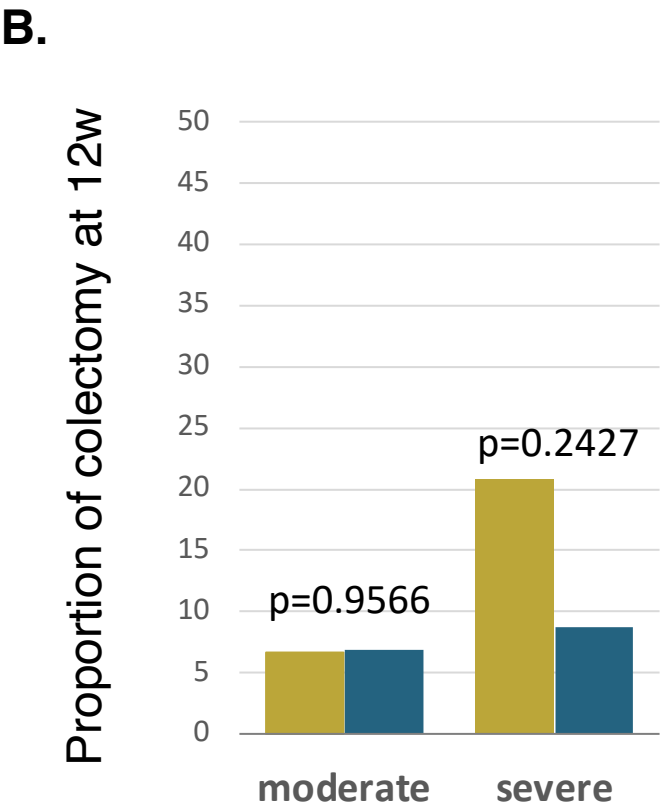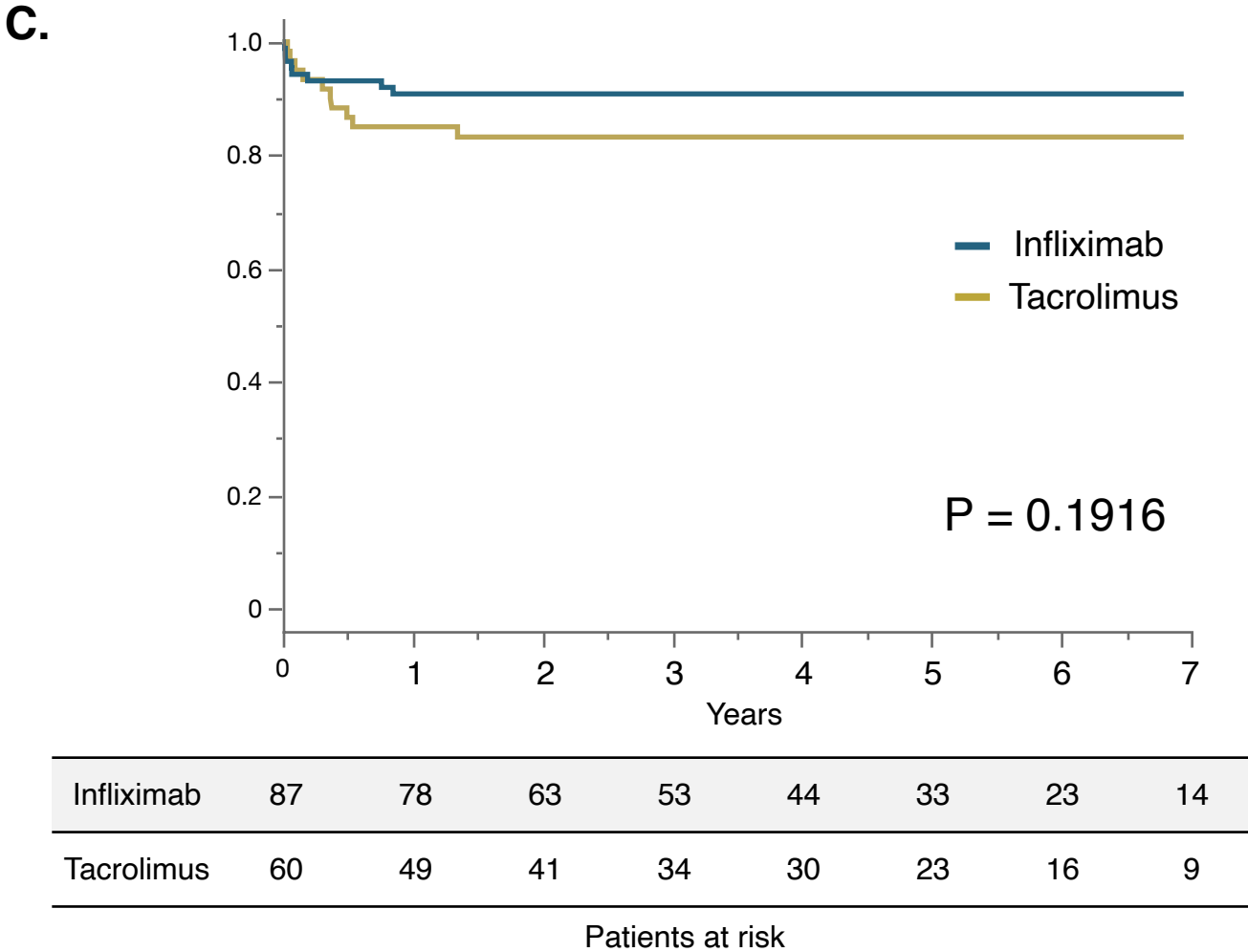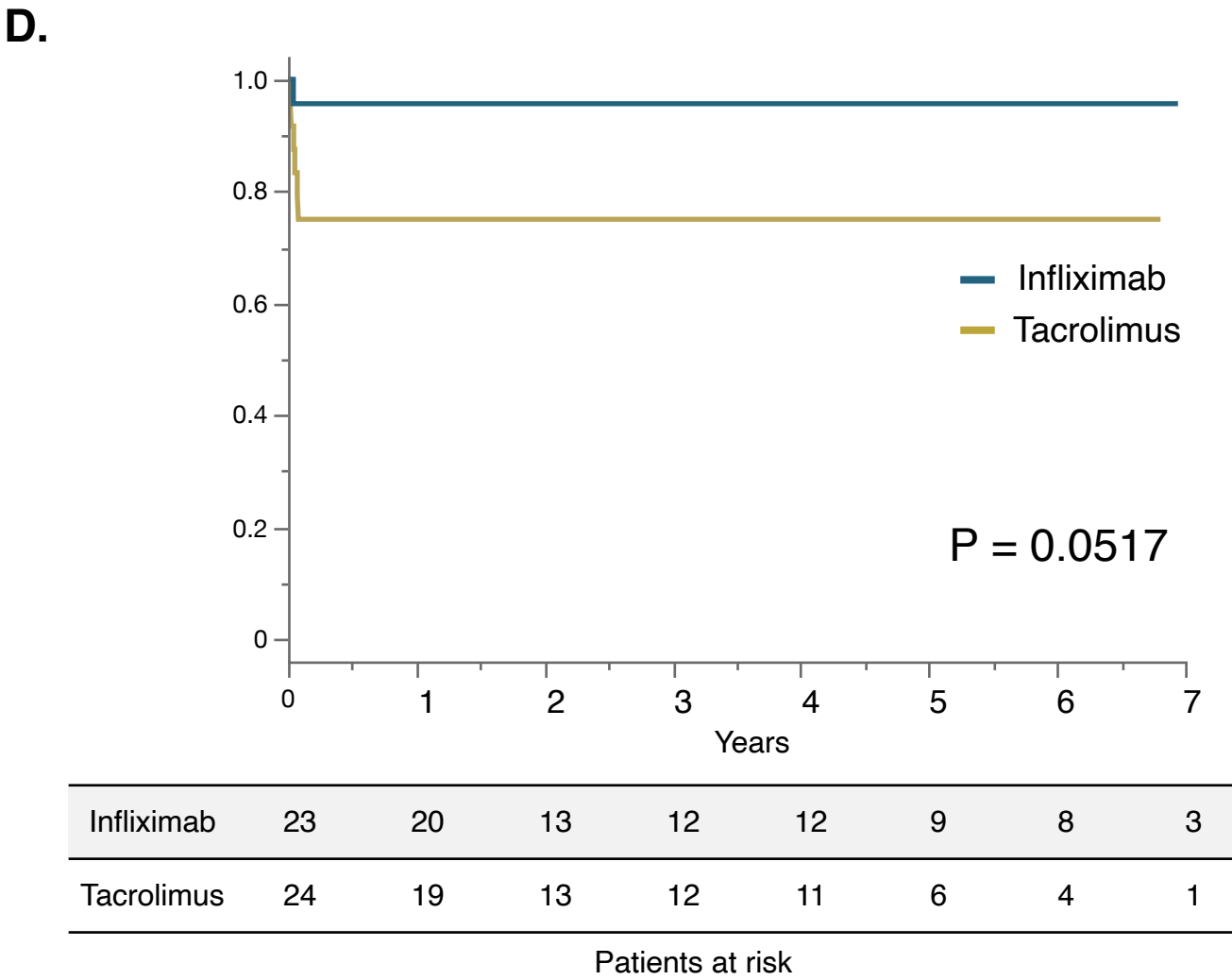

Supplement: Supplementary file 2 — Supplementary Information 2. [file 41598_2024_77365_MOESM2_ESM.pdf]

Colectomy-free survival rate

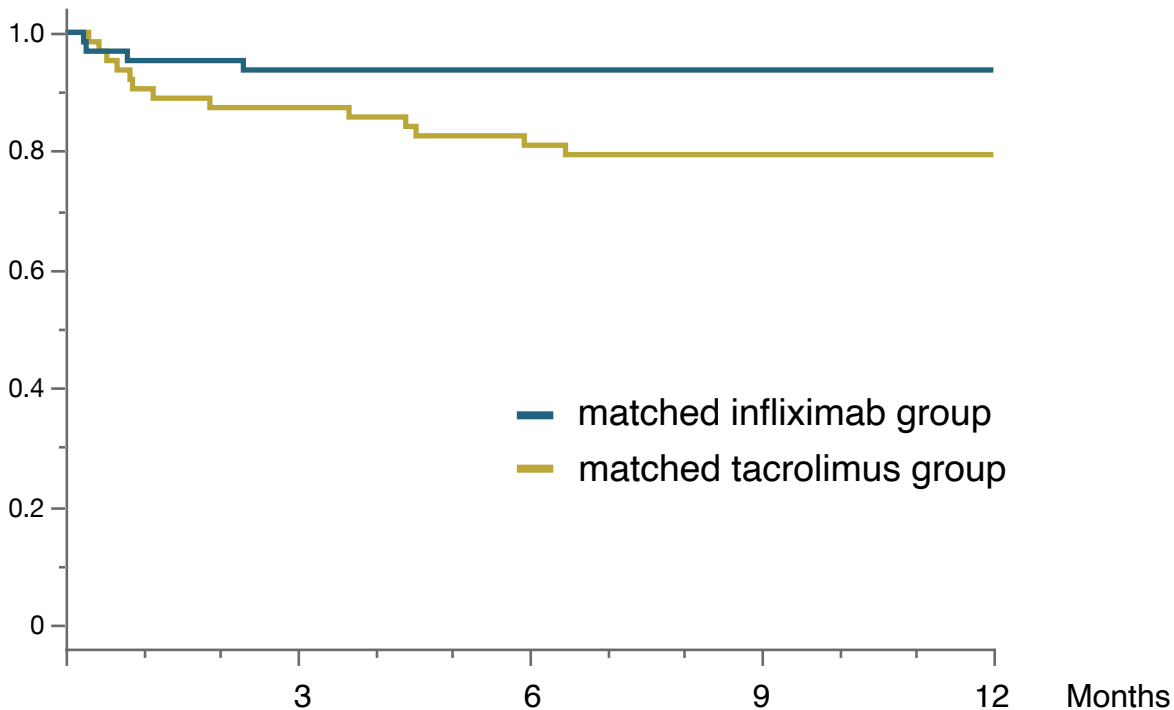

|            |    |    |    |    |    |
|------------|----|----|----|----|----|
| Infliximab | 63 | 60 | 59 | 59 | 59 |
| Tacrolimus | 63 | 56 | 52 | 50 | 48 |

Patients at risk

Supplement: Supplementary file 3 — Supplementary Information 3. [file 41598_2024_77365_MOESM3_ESM.pdf]
