# Supplementary material for: Effectiveness of tacrolimus therapy in refractory ulcerative colitis compared to infliximab with propensity score matching
Source: Sci Rep. 2025 Jan 2;15:68. doi: 10.1038/s41598-024-77365-y (PMC11696101; doi:10.1038/s41598-024-77365-y)
Supplement: Supplementary file 4 — Supplementary Information 4. [file 41598_2024_77365_MOESM4_ESM.pdf]

Supplementary Table 1. The List of ethics committees that approved the study**.**

**1**	The ethics committee of Osaka University Hospital
**2**	The ethics committee of Osaka Rosai Hospital
**3**	The ethics committee of National Hospital Organization Osaka National Hospital
**4**	The ethics committee of Toyonaka Municipal Hospital
**5**	The ethics committee of Osaka General Medical Center
**6**	The ethics committee of Osaka Police Hospital
**7**	The ethics committee of National Hospital Organization Osaka Minami Medical Center
**8**	The ethics committee of Kansai Rosai Hospital
**9**	The ethics committee of Ikeda City Hospital
**10**	The ethics committee of Itami City Hospital
**11**	The ethics committee of Higashiosaka City General Hospital
**12**	The ethics committee of Otemae Hospital
**13**	The ethics committee of Nishinomiya Municipal Central Hospital
**14**	The ethics committee of Japan Community Healthcare Organization Osaka Hospital
**15**	The ethics committee of NTT-West Osaka Hospital
**16**	The ethics committee of Hyogo Prefectural Nishinomiya Hospital
**17**	The ethics committee of Saiseikai Senri Hospital
**18**	The ethics committee of Yao Municipal Hospital

Supplementary Table 2. Patient characteristics of moderately active patients

	Tacrolimus group N=60	Infliximab group N=87	P value
Male, n (%)	36 (60)	52 (60)	n.s.
Age >60	16 (27)	22 (25)	n.s.
Disease duration (y) median [IQR]	2.0 [0.0-7.3]	2.0[1.0-7.0]	n.s.
BMI (kg/m2), median [IQR]	20.2 [18.1-22.9]	20.2 [17.9-22.9]	n.s.
Smoking, n (%)	14 (23)	23 (27)	n.s.
History of induction with steroids, n (%)	27 (45)	43 (49)	n.s.
Biologic / SMD naive	43 (72)	61 (70)	n.s.
Concomitant medications			
5-ASA, n (%)	57 (95)	79 (91)	n.s.
Steroids, n (%)	43 (72)	54 (62)	n.s.
Thiopurines, n (%)	14 (23)	38 (44)	0.014
Disease phenotype (pan-colitis), n (%)	49 (82)	57 (66)	0.040
Partial Mayo score, median [IQR]	6.5 [6-7]	6 [5-7]	n.s.
Mayo endoscopic subscore, (MES=3, Tac:20, IFX: 19)	25 (42)	31 (37)	n.s.
Hemoglobin (g/dl)	10.5 [9.4-12.3]	10.8 [9.8-12.8]	n.s.
Hemoglobin <10.5, n (%)	29 (48)	33 (38)	n.s.
CRP (mg/dl), median [IQR]	2.5 [0.5-4.2]	0.6 [0.2-2.2]	0.001
CRP >3.0mg/dl, n (%)	27 (45)	19 (22)	0.004
Hemoglobin <10.5 or CRP >3.0mg/dl, n (%)	39 (65)	42 (48)	0.045
Albumin (g/dl), median [IQR]	2.9 [2.3-3.4]	3.3 [2.7-3.8]	0.006
Albumin <3.5 g/dl, n (%)	32 (56)	39 (46)	n.s.

IQR: interquartile range, BMI: body mass index, SMD: small molecule drug, ASA: aminosalicylic acid, CRP: c-reactive protein, n.s.: not significant

Supplementary Table 3. Patient characteristics of severely active patients

	Tacrolimus group N=24	Infliximab group N=23	P value
Male, n (%)	12 (50)	15 (65)	n.s.
Age >60	1 (4)	4 (17)	0.023
Disease duration (y) median [IQR]	5.0 [0.8-9.3]	2.0[0.0-7.0]	n.s.
BMI (kg/m2), median [IQR]	21 [18-22]	20 [18-21]	n.s.
Smoking, n (%)	6 (25.0)	6 (26.1)	n.s.
History of induction with steroids, n (%)	8 (33)	14 (61)	0.082
Biologic / SMD naive	22 (92)	19 (83)	n.s.
Concomitant medications			
5-ASA, n (%)	23 (96)	21 (91)	n.s.
Steroids, n (%)	13 (54)	17 (74)	n.s.
Thiopurines, n (%)	5 (21)	8 (35)	n.s.
Disease phenotype (pan-colitis), n (%)	17 (70)	19 (83)	n.s.
Partial Mayo score, median [IQR]	8 [8-9]	8 [8-9]	n.s.
Mayo endoscopic subscore, (MES=3, Tac:20, IFX: 19)	20 (100)	17 (74)	n.s.
Hemoglobin (g/dl)	11.8 [9.8-12.7]	10.8 [9.6-11.6]	n.s.
Hemoglobin <10.5, n (%)	7 (29)	9 (39)	n.s.
CRP (mg/dl), median [IQR]	4.8 [3.4-6.9]	3.5 [0.2-6.0]	n.s.
CRP >3.0mg/dl, n (%)	18 (75)	12 (52)	n.s.
Hemoglobin <10.5 or CRP >3.0mg/dl, n (%)	21 (88)	13 (57)	0.024
Albumin (g/dl), median [IQR]	2.8 [2.6-3.4]	3.2 [2.1-3.6]	n.s.
Albumin <3.5 g/dl, n (%)	12 (50)	10 (46)	n.s.

IQR: interquartile range, BMI: body mass index, SMD: small molecule drug, ASA: aminosalicylic acid, CRP: c-reactive protein, n.s.: not significant

Supplementary Table 4. Patient characteristics in the tacrolimus/non-Biologics group and the tacrolimus/Biologics group

	Tacrolimus/non-Biologics group	Tacrolimus/Biologics group	P value
All, n (%)	41	17	
Male, n (%)	25 (61)	10 (59)	n.s.
Age (y), median [IQR]	42 [33-59]	51 [31-63]	n.s.
Disease duration (y), median [IQR]	3.0 [0-10]	3.0 [0-9.5]	n.s.
BMI (kg/m2), median [IQR]	20.3 [18.5-22.8]	21.9 [18.2-24.7]	n.s.
Smoking, n (%)	10 (24)	7 (41)	n.s.
History of induction therapy with steroids, n (%)	13 (32)	10 (58)	n.s.
Biologic / SMD naive	36 (88)	13 (76)	n.s.
Concomitant medications at 0w			
5ASA, n (%)	38 (93)	16 (94)	n.s.
Steroids, n (%)	30 (73)	10 (59)	n.s.
Thiopurines, n(%)	9 (22)	4 (24)	n.s.
Disease phenotype (pan-colitis), n (%)	35 (85)	14 (82)	n.s.
partial Mayo score at 0w, median [IQR]	7 [6-8]	7 [4.5-8]	n.s.
Mayo endoscopic subscore at 0w, median [IQR]	2.5 [2-3]	3 [2-3]	n.s.
Disease severity (Mild/Moderate/Severe)	4/28/9	1/11/5	n.s.
CRP at 0w (mg/dl), median [IQR]	3.31 [1.22-6.12]	2.57 [0.49-5.76]	n.s.
Albumin at 0w (g/dl), median [IQR]	2.6 [2.2-3.2]	3.1 [2.3-3.8]	n.s.
partial Mayo score at 8w, median [IQR]	0 [0-1]	1 [1-2]	0.0363
CRP at 8w (mg/dl), median [IQR]	0.12 [0.04-0.34]	0.06 [0.03-0.70]	n.s.
Albumin at 8w (g/dl), median [IQR]	4.0 [3.7-4.4]	4.2 [3.9-4.4]	n.s.
Duration of tacrolimus therapy, median [IQR]	3.0 [2.0-6.8]	3.0 [2.0-7.5]	n.s.
Biologics in maintenance phase (IFX/ADA/GLM/VED)	-	10/3/2/2	n.s.
Concomitant thiopurines in maintenance phase, n (%)	29 (71)	9 (53)	n.s.

IQR: interquartile range, BMI: body mass index, SMD: small molecule drug, ASA: aminosalicylic acid, CRP: c-reactive protein, n.s.: not significant

Supplementary Table 5. Patient characteristics at baseline in the tacrolimus/non-Biologics group between patients with or without colectomy

	Colectomy	Colectomy-free	P value
All, n (%)	6	35	
Male, n (%)	5 (83)	20 (57)	n.s.
Age (y), median [IQR]	59 [31-72]	42 [33-58]	n.s.
Disease duration (y), median [IQR]	1.5 [0-2.8]	5 [1.0-10]	n.s.
BMI (kg/m2), median [IQR]	19.1 [17.6-20.4]	21.1 [18.5-23.0]	n.s.
Smoking, n (%)	1 (17)	9 (25.7)	n.s.
History of induction therapy with steroids, n (%)	1 (17)	12 (34)	n.s.
Biologic / SMD naive	5 (83)	31 (89)	n.s.
Concomitant medications			
5ASA, n (%)	6 (100)	32 (91)	n.s.
Steroids, n (%)	6 (100)	24 (69)	n.s.
Thiopurines, n (%)	1 (17)	8 (23)	n.s.
Disease phenotype (pan-colitis), n (%)	6 (100)	29 (83)	n.s.
partial Mayo score, median [IQR]	6 [4-7]	7 [6-8]	n.s.
Mayo endoscopic subscore, median [IQR]	2.5 [1.75-3]	2.5 [2-3]	n.s.
Disease severity (Mild/Moderate/Severe)	1/5/0	3/23/9	n.s.
CRP at 0w (mg/dl), median [IQR]	4.36 [2.60-5.95]	2.77 [1.07-6.14]	n.s.
Albumin at 0w (g/dl), median [IQR]	2.2 [2.0-2.9]	2.6 [2.3-3.2]	n.s.

IM: immunomodulator, IQR: interquartile range, BMI: body mass index, SMD: small molecule drug, ASA: aminosalicylic acid, CRP: c-reactive protein, n.s.: not significant
